# Biocuration of functional annotation at the European nucleotide archive

**DOI:** 10.1093/nar/gkv1311

**Published:** 2015-11-28

**Authors:** Richard Gibson, Blaise Alako, Clara Amid, Ana Cerdeño-Tárraga, Iain Cleland, Neil Goodgame, Petra ten Hoopen, Suran Jayathilaka, Simon Kay, Rasko Leinonen, Xin Liu, Swapna Pallreddy, Nima Pakseresht, Jeena Rajan, Marc Rosselló, Nicole Silvester, Dmitriy Smirnov, Ana Luisa Toribio, Daniel Vaughan, Vadim Zalunin, Guy Cochrane

**Affiliations:** European Molecular Biology Laboratory, European Bioinformatics Institute (EMBL-EBI), Wellcome Genome Campus, Hinxton, Cambridge, CB10 1SD, UK

## Abstract

The European Nucleotide Archive (ENA; http://www.ebi.ac.uk/ena) is a repository for the submission, maintenance and presentation of nucleotide sequence data and related sample and experimental information. In this article we report on ENA in 2015 regarding general activity, notable published data sets and major achievements. This is followed by a focus on sustainable biocuration of functional annotation, an area which has particularly felt the pressure of sequencing growth. The importance of functional annotation, how it can be submitted and the shifting role of the biocurator in the context of increasing volumes of data are all discussed.

## INTRODUCTION

The European Nucleotide Archive (ENA), maintained at the European Bioinformatics Institute (EMBL-EBI) in Hinxton, UK, is a permanent nucleotide sequencing repository which hosts the world's DNA and RNA sequences, along with associated metadata, functional annotation and other derived data. Its focus lies in the capture, standardisation and organisation of sequencing data, maintenance and storage of the data and presentation of the data to the scientific community. Groupings of data types within ENA, called *domains*, allow for easy access and discoverability (1). For example, raw data can be accessed through the *Read domain*; assembled and (optionally) annotated reads can be accessed through the *Sequence domain*. A full description of ENA domains is provided at URL: http://www.ebi.ac.uk/ena/submit/data-formats.

Public data are exchanged daily between members of the International Nucleotide Sequence Database Collaboration (INSDC (2)), which comprises the ENA and its partners, the National Center for Biotechnology Information (NCBI GenBank (3)) and the DNA Databank of Japan (DDBJ (4)). Under this long-standing partnership, we ensure the fullest reach of archived sequence data and participate in the development of global standards for data collection and management. The taxonomic classification in use at ENA is the NCBI Taxonomy (5). The systematic range of taxa spans the complete taxonomic realm from viruses to eukaryotes, with nomenclature for describing unpublished and unknown taxa, as well as mixed libraries, metagenomic samples and synthetic molecules.

In this article, we describe the continuing growth of sequencing data in the ENA, with some notable data sets that have been released over the last year, in the fields of pathogen surveillance, genomics, conservation, anthropology and marine science. Major achievements are then reviewed in brief, followed by a more in-depth look at functional annotation and the changing role of the ENA biocurator.

## CONTENT AND GROWTH

ENA continues to see exponential growth in data. The *Sequence* and *Read domains* are currently seeing similar doubling rates of 22.8 months and 21.8 months respectively, with a total of 622.7 million assembled sequences and 15.8 trillion reads. The total number of bases in ENA at the time of writing in ENA has exceeded 2.07 × 10^15^ and covers over 365 000 formally described species and infra-species and over 1.2 million taxa. Further statistical information, which is updated on a weekly basis, can be accessed here: http://www.ebi.ac.uk/ena/about/statistics.

ENA hosts many important and notable data sets, which have been published during the previous year. In January 2015, RNA-Seq libraries were released for six UK samples of the fungus *Hymenoscyphus fraxineus* (ENA accession PRJEB7998), a pathogen causing Ash Dieback disease, which is still a major concern in Europe. In March/April 2015, 131 *Influenza* genomes (ENA accession PRJEB8717) were submitted through the ENA's genome assembly pipeline, thereby publicly providing the supporting data from a study into the flu pandemic, which had emerged in 2009 (6). In May 2015, INRA, in partnership with Genoscope, released the first paper (7) and draft genome for the iconic oak species, *Quercus ruber* (ENA accession PRJEB7855). The assembled and annotated draft genome sequence of the North Island brown kiwi (*Apteryx mantelli*; ENA accession PRJEB6383; http://www.mpg.de/research/kiwi-bird-genome-sequenced), a ground-dwelling, nocturnal ratite of New Zealand, was released in June 2015 (8). In the same month, Guerrini and co-workers published an article on the Cypriot Mouflon (including ENA accessions LN651259-LN651268), to challenge its controversial status as a subspecies of domestic sheep (*Ovis aries*), a fact which is hampering conservation issues for this endangered bovid (9).

In August 2015, The Pääbo Lab published sequencing data (ENA accession PRJEB8987) from an early modern human, alive some 37 000–42 000 years ago, which was found to have a genetic contribution from Neanderthal many times that of the present modern population, suggesting a very recent Neanderthal ancestor (10). Another publication in September 2015, also on the theme of ancient DNA, has uncovered clues to the demographic origins of the Basque people of northern Spain and southern France (11). Genome sequencing data from eight prehistoric skeletons from Atapuerca in Spain (ENA accession PRJEB9783) revealed an admixed gene pool of early farmer with local hunter-gatherer showing greatest affinity to modern day Basques. This strongly suggests a Basque origin in the spread of farming during the Neolithic and their genetic isolation from later human migrations that affected the remaining modern-day Iberian groups.

The analyses of the largest DNA sequencing initiative in ocean science (Tara Oceans; ENA accession PRJEB402) were published back-to-back in a special issue of Science in May 2015 (12). These analyses revealed the largely unknown biodiversity of planktonic communities and their interactions within temperature-dependent ecosystems. Derived data are also available through ENA, such as that from the metagenomic pan-gene analysis (ENA accession PRJEB7988). Also with the marine theme: Ocean Sampling Day is a simultaneous sampling for marine microorganisms in a global network of over 150 marine stations covering all continents. Marine microorganisms have been collected according to standardised sampling protocols during the summer solstices on June 21st in the years 2014 and 2015. Richly and consistently described samples from this high profile study (ENA accession PRJEB5129) are now available in the ENA together with raw amplicon and shotgun metagenomes from the 2014 sampling (ENA accession PRJEB8682) and derived data processed by EBI Metagenomics (ENA accession PRJEB9694).

## GENERAL ACHIEVEMENTS

### Tabulated programmatic submissions

A new programmatic tabulated submission service was fully introduced in July 2015. This system is provided in addition to the existing XML-based programmatic submission service and allows the provision of metadata using spreadsheets rather than constructed XMLs. Full documentation is available via the ENA website (http://www.ebi.ac.uk/ena/submit/programmatic-tab-submission).

### Assembly pipeline

Since the genome assembly submission tool was launched in April 2014, it has seen considerable uptake by the scientific community. Recent developments in 2015 have been aimed at accepting virus genomes through the pipeline. This development now provides bulk and programmatic submitters of virus genomes the same simplicity and ease of use that has previously been limited to prokaryotic and eukaryotic genome submitters. Note that plasmid and organellar genomes are still not currently accepted unless submitted with accompanying chromosomes.

### CRAM

The CRAM framework technology for reference-based compression of sequence data has been enhanced to version 3 (http://www.ebi.ac.uk/ena/software/cram-toolkit). CRAM 3 comes with many advantages over the previous release (2.1): faster and more effective compression, adoption of new codecs (rANS codec, xz, bzip2), addition of checksums in container headers and blocks, optimised representation of bases and scores, support for unsorted data, support for container-specific embedded reference sequence fragments and support for end of file (EOF) marker. The CRAM Reference Registry (http://www.ebi.ac.uk/ena/software/cram-reference-registry), a service for the unambiguous retrieval of reference sequences by their md5 checksum, has been improved to better support the format's use across all read submissions into ENA. The CRAM reference registry now contains all assembled INSDC sequences and is updated on a daily basis. In addition, the CRAM reference registry accepts direct reference sequence submissions from sequencing centres to allow read submissions in CRAM format in cases where the reference sequences themselves have not yet been submitted into the INSDC.

### Cloud delivery

ENA has continued to make investments in the delivery of data through cloud technology. So far, this work has used the existing EMBL-EBI Embassy infrastructure and leveraged ENA's recent move from conventional file storage to an object store system. This system supports much simpler management operations and offers greater flexibility in supporting users and data endpoints. Infrastructure has been deployed enabling data tagging (based on curated rules) and flow of tagged data to a defined data cache and presentation endpoint. Specifically, cache layers in a number of Embassy instances have been generated for use by the Tara Oceans consortium (the ‘marine cache’) and the COMPARE community (the ‘pathogen cache’).

### Cross-references

Traditionally, ENA has supported cross-references to external resources for records in the *Sequence* and *Coding domains* only. Cross-references were updated during quarterly *Sequence* release, and written into flat files and made available in the ENA browser as links from a record. Support for cross-references has been expanded over the last two years to include records across all ENA domains and to allow for a more frequent update cycle. While there are still limitations preventing the update of cross-references in flat files outside of the quarterly release cycle, all cross-references available in the ENA browser are kept up-to-date.

In 2014, ENA introduced a programmatic service for users to obtain the latest cross-reference data (described at http://www.ebi.ac.uk/ena/browse/xref-service-rest). During 2015, this service was expanded into its own search service available from the browser (http://www.ebi.ac.uk/ena/data/xref/search), allowing users to:

1. Find and download the full set of cross-references for any external source data resource,
2. Locate all records at ENA that reference a given external record and
3. Retrieve all cross-references for an ENA data record.

### Biocuration and Checklists

In December 2014, ENA biocurators along with eight external biological researchers formed a one-week sample record annotation jamboree. The aim was to annotate richly a selection of environmental sample records with particular focus on ontologies such as the Environment Ontology (EnvO, http://environmentontology.org). Nearly 2000 public records were successfully enriched, thereby providing better usability and retrieval for ENA users, as well as doubling the number of samples which passed a quality threshold for downstream analysis by EMBL-EBI Metagenomics (EMG; https://www.ebi.ac.uk/metagenomics/).

Sample Checklist development has continued in 2015 (http://www.ebi.ac.uk/ena/submit/checklists). Checklists for capturing sample metadata of general virus pathogens, and also specifically for the influenza virus, have been released in support of the COMPARE project, an EU initiative of pathogen surveillance using genome technology. The INSDC agreed in 2015 to the use of a missing value vocabulary, enabling submitters to adhere to standards where certain metadata values are either inappropriate, not collected, to be provided at a later date, or strictly confidential due to privacy concerns (http://www.ebi.ac.uk/ena/about/missing-values-reporting).

## FUNCTIONAL ANNOTATION

### Introduction

Feature annotation on records in the ENA *Sequence domain* is mandatory and is governed by the INSDC Feature Table, a framework for describing locations and features of the biological source and higher order genetic features on nucleic acids (http://www.insdc.org/files/feature_table.html). The definitions, conditions and usage of the feature keys (hereafter referred to as ‘features’) and their qualifiers are also publicly available here: http://www.ebi.ac.uk/ena/WebFeat/. The minimal feature annotation in any record is a *source* feature although most record types require additional functional annotation, which is further described below. The *source* feature must contain the organism name (/organism) and the molecule type which was sequenced (/mol_type). The organism name is controlled by the INSDC reference taxonomic database NCBI Taxonomy (http://www.ncbi.nlm.nih.gov/taxonomy/) and may be either a biological organism or a synthetic molecule; rules are in place for naming environmental samples, mixed libraries and unpublished or unidentified organisms. As well as /organism and /mol_type, the *source* feature may contain other qualifiers which record further organismal (e.g. /cultivar), sample (e.g. /tissue_type) and experimental (e.g. /PCR_primers) metadata, thereby sharing many attributes with the sample and experiment records, familiar for submitters of *Read domain* data. In fact, submissions of genome assemblies into ENA require generation or re-use of *Sample* records through Sample Checklists, the information from which is then propagated onto the *source* feature of *Sequence domain* records (WGS sets, chromosome records, etc.)

Functional annotation has always been a key aspect in *Sequence* records. This specific type of annotation describes the genetic features upon the submitted sequence. Those records with functional annotation are often described as being ‘annotated’ whereas those without are ‘unannotated’. Traditionally, only certain types of *Sequence* records were allowed to exist without functional annotation; WGS (Whole Genome Shotgun), CON (Constructs) and TSA (Transcriptome Shotgun Assembly) dataclass records may be either annotated or unannotated; EST (Expressed Sequence Tag) and GSS (Genome Survey Sequence) never contain functional annotation. In the STD (Standard) dataclass, only featureless viroids were exempt from annotation. However, since 2013, due to the overwhelming increase in multi-isolate genome sequencing projects, the INSDC agreed to allow genome records, at any stage of assembly (WGS contigs, CON scaffolds, STD replicons), to be submitted with or without functional annotation; where a reference genome is unavailable it is still highly recommended to submit functional annotation.

**Figure 1.**
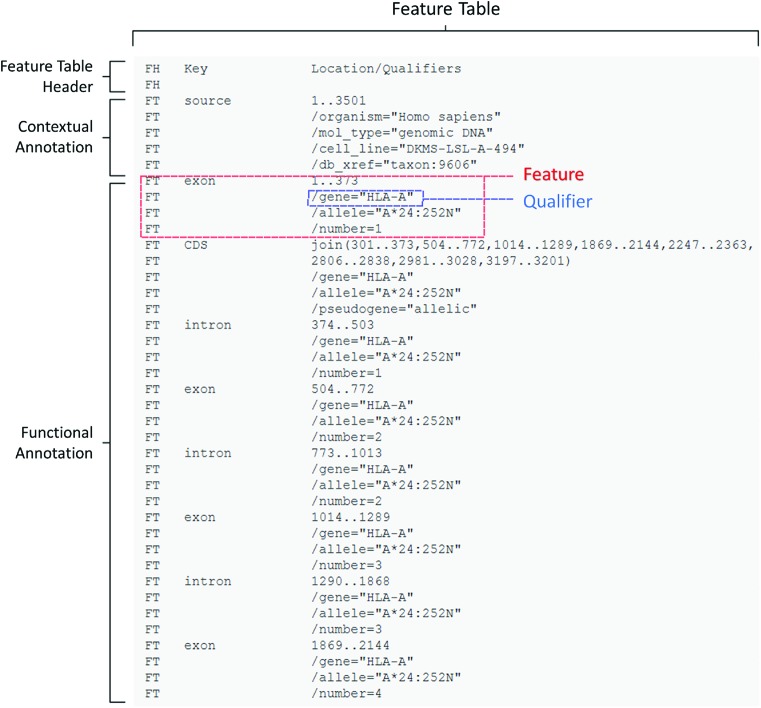
Example of Feature Table annotation for an HLA-A gene (taken from ENA accession LN873232). See the text for details.

An example of feature annotation in ENA flat file format for an HLA-A gene allele is shown in Figure 1. The full flat file record can be seen at http://www.ebi.ac.uk/ena/data/view/LN873232&display=text and the default browser view is available at http://www.ebi.ac.uk/ena/data/view/LN873232, with tabs leading to ‘Source feature(s)’ and ‘Other feature(s)’, the latter being for functional annotation. In flat file format, the Feature Table begins with a header (*FH* lines), followed by the feature table annotation (*FT* lines). The mandatory *source* feature begins the table and describes, using qualifiers and their values, the source organism, the molecule sequenced and the laboratory cell line. The functional annotation follows for the components of a well-defined HLA-A gene. *Exon*, *intron* and *CDS* features are all provided with their relative coordinates within the archived sequence. Each of the features is individually labelled with qualifier values that describe the gene and allele names. In addition, *exon* and *intron* features contain numbering (*/number*) and the *CDS* feature captures the null allele status of this particular variant (*/pseudogene*).

### Importance

The benefit of functional annotation is great. Users want to know what genetic features lie within the sequences in the database, where exactly they are located and what characteristics they have. Without such annotation, the user of a *Sequence* record would have to perform their own inferential analysis or experimentation to obtain information that may have been known by the depositor of the record at the time of submission. Direct access to views of functional annotation can be gained via the ENA Browser (www.ebi.ac.uk/ena/data/view/{accession}) where the user can visualise a feature or set of features in context. A typical user may access *Sequence* records for this kind of visualisation through sequence homology tools such as ENA Search (www.ebi.ac.uk/ena/data/sequence/search); here the user can map functional annotation from search results directly onto their own query sequence via the resulting alignment. In other situations, a user may reach a record through the ENA's Advanced search tool, which allows combinations of features, qualifiers and their values to be searched. Such searches are effected using the *Coding*, *Non-coding* (see below) and *Marker domains* (for a selection of commonly used phylogenetic marker loci).

Annotated *Sequence* records are exported into other ENA domains for use by individual researchers, specialist communities and downstream databases. For example, coding sequences are captured using the *CDS* feature and non-coding loci by a myriad of features including *rRNA*, *tRNA* and *ncRNA*. Each feature may contain a wealth of additional information captured in qualifiers; these may describe the product name (/product), the gene symbol (/gene) or locus tag (/locus_tag), any special conditions (e.g. /ribosomal_slippage or /trans_splicing), categorisation of feature (e.g. /ncRNA_class) and supporting evidence (e.g. /experiment or /inference). The *CDS* feature is translated based on the systematic range of the source organism and a unique protein identifier (/protein_id) is provided that has been generated by ENA upon submission. The complete *CDS* feature, with accompanying *source* information and nucleotide sequence, is extracted into its own record for use in the *Coding domain*. Similarly, non-coding type features are systematically extracted into the *Non-coding domain*. Both *Coding* and *Non-coding domains* are fully searchable using ENA's Advanced Search (http://www.ebi.ac.uk/ena/data/warehouse/search), use their own accessions allowing easy citation and are also directly consumed by resources such as UniprotKB (http://www.uniprot.org/) and RNAcentral (http://rnacentral.org/), which give their users expert classification and further analysis in addition to reciprocal access back to the original records in ENA.

Functional annotation is also very important to capture in cases where wet labs have experimentally verified nucleotide features and their characteristics, such as splice variants and allele designations. Although the majority of annotation on a feature-frequency basis is coming from automated gene predictions on genome assemblies, it is often the small-scale submitter, working in a research lab and focused on a particular gene or marker of interest, who can provide the best biologically-supported annotation. Such details when captured on the submitted nucleotide records will then aid downstream users and will ultimately lead to better gene models as they serve as a foundational reference set for annotation inference processes. INSDC feature qualifiers which describe experimental support include /citation (refers to a specific reference within the record for that feature) and /experiment (a free text attribute which can be categorised by support for the feature coordinates, function or existence).

### Submission and validation

The submission system of *Sequence* records has evolved substantially over the last six years. We have undergone a transition from predominantly manual biocuration, at high time cost, to more automated systems based on curated ‘Annotation Checklists’ and validation schemes built from curated annotation rules. Data submissions to ENA are routed through the Webin system (http://www.ebi.ac.uk/ena/submit). Submission case-specific documentation is available from http://www.ebi.ac.uk/ena/submit/sequence-submission.

The Annotation Checklists (currently 34 in number) serve to assist in the submission of the most common types of *Sequence domain* records (see Table 1). Designed to allow the user to submit data without needing to know the Feature Table language, the checklists are presented, according to the user's preference, as web forms within Webin or as pro-forma spreadsheets for offline data entry and subsequent upload. Each checklist includes a set of mandatory and user-optional fields, which guide the user and ultimately populate the final underlying feature annotation. The checklists bring annotation consistency within data sets and avoid conflicts of Feature Table syntax. For unusual or complex feature annotation, an ‘Entry Upload’ route is provided. Here the user is required to build the Feature Table, either manually or by means of external genome annotation software (such as Artemis (13)), and upload it directly into Webin. ‘Template’ examples and detailed documentation are provided to support users who are less familiar with the INSDC Feature Table language (http://www.ebi.ac.uk/ena/submit/entry-upload-templates).

**Table 1.** Annotation Checklists currently available for submitting simple annotations and marker sequences

Type	Name	Description
Frequently used	rRNA gene	For ribosomal RNA genes from prokaryotic, nuclear or organellar DNA. All rRNAs are considered partial.
	Single CDS genomic DNA	For complete or partial coding sequence (CDS) derived from genomic DNA. This checklist will not accept segmented genes (i.e. with intron regions) so should be used for prokaryotic, organellar genes or for submitting a single exon.
	Single CDS mRNA	For complete or partial single coding sequence (CDS) derived from mRNA (via cDNA). Do NOT use for submission of VIRTUAL transcripts (TSA or Unigene clusters)—use TSA CDS Annotated checklist.
	Multi-Exon Gene	For the submission of single complete or partial multi-exon genes from *in vivo* genomic DNA. This checklist captures the gene region but does not capture exon, intron or CDS features. No translation will be generated. If precise annotation or translation is required, please use an alternative submission route. Single exon genes or sequences covering a single exon only from a multi-exon gene can be submitted using the Single CDS Genomic DNA checklist. For HLA/MHC genes which cannot be submitted using ‘MHC gene 1 exon’ or ‘MHC gene 2 exon’ checklist, please use the Entry Upload option (see: http://www.ebi.ac.uk/ena/submit/entry-upload-templates)
	MHC gene 1 exon	For partial MHC class I or II antigens containing one exon ONLY.
	MHC gene 2 exons	For partial MHC class I or II antigens containing two exons ONLY. An intron feature should only be used when the intron region has actually been sequenced. If the intron has not been sequenced, or only partially sequenced, please fill the non-sequenced gap with 100 Ns.
	ncRNA	For non-coding RNA (ncRNA) transcripts or single-exon genes of prokaryotic or eukaryotic origin with the exception of the ribosomal RNA (rRNA) and transfer RNA (tRNA).
	Satellite DNA	For submission of Satellites, Microsatellites and Minisatellites. Complete or partial single polymorphic locus present in nuclear and organellar DNA that consists of short sequences repeated in tandem arrays.
	Mobile Element	For the submission of a single complete or partial mobile element. This checklist captures the mobile element feature but does not allow for granular annotation of component parts, such as coding regions, repeat regions and miscellaneous features within the mobile element itself. If precise annotation or translation is required, please use an alternative submission route.
	Gene Promoter	For submission of uni- or bi-directional gene promoter regions. Please note that CDS is not annotated; if you wish to include the start of the coding region(s), please leave a comment with the coordinates of the start site(s).
Marker sequence	COI gene	For mitochondrial cytochrome oxidase subunit 1 genes.
	ITS rDNA	For ITS rDNA region. This checklist allows generic annotation of the ITS components (18S rRNA, ITS1, 5.8S rRNA, ITS2 and 28S rRNA). For annotation of the rRNA component only, please use the rRNA gene checklist.
	trnK-matK locus	For complete or partial matK gene within the chloroplast trnK gene intron.
	Phylogenetic Marker	For the submission of the following markers: actin (act), tubulin (tuba or tubb), calmodulin (CaM), RNA polymerase II large subunits (RPB1 and RPB2), translation elongation factor 1-alpha (tef1a), glyceraldehyde 3-phosphate dehydrogenase (GAPDH) and histone 3 (H3) where the intron/exon boundaries are not known.
	Multi-Locus Marker	For the submission of multi-locus markers (e.g. tRNA + CDS + rRNA) from *in vivo* gemomic DNA. This checklist provides a simple submission process for organellar or nuclear regions containing multiple genes. For example, a region containing coding genes, rRNA genes and tRNA genes. Please note that individual feature annotation is not possible with this checklist.
	D-Loop	For mitochondrial D-loop (control region) sequences. All D-loops are considered partial.
	Intergenic Spacer, IGS	For intergenic spacer (IGS) sequences between neighbouring genes (e.g. psbA-trnH IGS, 16S-23S rRNA IGS). Inclusion of the flanking genes is allowed.
	Gene intron	For complete or partial single gene intron.
	External Transcribed Spacer (ETS)	For submission of External Transcribed Spacer (ETS) regions of the eukaryotic rDNA transcript; a region often used to study intrageneric relationships.
	16S-23S Intergenic Spacer Region	For submission of the 16S-23S rRNA intergenic spacer region: the transcribed spacer between the 16S rR NA and 23S rRNA genes of rRNA operons, found in prokaryotes and organelles.
Virus-specific	Single Viral CDS	For complete or partial single coding sequence (CDS) from a viral gene. Please do not use for peptides processed from polyproteins or proviral sequences, as these are all annotated differently.
	Viral Polyprotein	For complete or partial viral polyprotein genes where the mature peptide boundaries remain undefined. This template is not suitable for proviral sequences. If the sequences contain ribosomal frameshifts, please contact us.
	ssRNA(-) Viral copy RNA	For complete or partial viral copy RNA (cRNA) sequences, complementary to ssRNA(-) virus genomes. Only one CDS can be added; further CDS information should be provided in the curator comments section.
	Viral Untranslated Region (UTR)	For complete or partial untranslated region (UTR) or nontranslated region (NTR) found at the termini of viral genomes. Please do not use this checklist for submitting virus genomes or viral coding genes.
	Alphasatellite sub-viral particle	For submission of circular single stranded DNA alphasatellite sequences associated with Begomovirus, Babuvirus and Nanovirus.
	Betasatellite sub-viral particle	For submission of circular single stranded DNA betasatellite sequences of the Begomovirus genus.
	Plant Viroid	For complete circular ssRNA plant viroid sequences. Please do not use for other circular viruses.
Standards-Compliant	BARCODE COI	For Metazoan mitochondrial cytochrome oxidase subunit 1 (COI) genes that provide unique species-level identification and conform to Consortium for the Barcode of Life (CBoL) standards.
	GSC MIMARKS-Survey 16S rRNA sequences	For the submission of 16S rRNA (gDNA) sequences compliant with the GSC MIMARKS 4.0 standard. Users of this checklist must first submit their samples here: https://www.ebi.ac.uk/ena/submit/sra/#home
Large-scale data	Expressed Sequence Tag (EST)	For submission of Sanger-sequenced Expressed Sequence Tags (ESTs). ESTs are short transcripts ≈500–800 bp long usually of low quality as they are the result of only single pass reads. No feature annotation is recorded on ESTs.
	Sequence Tagged Site (STS)	For submission of Sequence Tagged Sites (STS). The Sequence Tagged Site (STS) is a relatively short, easily PCR-amplified sequence (200–500 bp) which can be specifically amplified by PCR and detected in the presence of all other genomic sequences and whose location in the genome is mapped.
	Genome Survey Sequence (GSS)	For submission of Genome Survey Sequences (GSS). These are short DNA sequences which inlude: random single pass genome survey sequences, single pass reads from cosmid/BAC/YAC ends (may be chromosome specific), exon trapped genomic sequences, Alu PCR sequences and transposon-tagged sequences.
	Transcriptome Shotgun Assembly (TSA)—Unannotated	For submission of virtual transcript assemblies (TSA, EST clusters) without feature annotation. IMPORTANT INFORMATION: virtual transcripts can ONLY be hosted with supporting evidence from raw experimental data. The raw reads should therefore be submitted to Read domain prior to the assembly being submitted as well as an alignment BAM file demonstrating how the raw reads are mapped to the transcripts. Please email datasub@ebi.ac.uk for further clarification.
	Transcriptome Shotgun Assembly (TSA)—CDS Annotated	For submission of virtual transcript assemblies (TSA, EST clusters) with CDS annotation. IMPORTANT INFORMATION: virtual transcripts can ONLY be hosted with supporting evidence from raw experimental data. The raw reads should therefore be submitted to the Read domain prior to the assembly being submitted as well as an alignment BAM file demonstrating how the raw reads are mapped to the transcripts. Please email datasub@ebi.ac.uk for further clarification.

More complex annotation should be submitted in an ENA-format flat file using ‘Entry Upload’ option.

In order to verify the metadata and functional annotation received with nucleotide sequences, all *Sequence* submissions are processed automatically through the ENA Validator prior to assignment of accession numbers. The Validator employs a set of curated rules, which check the syntax and semantics of the submitted sequence records. Core syntactic validation includes checking the usage, structure and interdependencies defined by the Feature Table language. For example, the qualifier /strain may not exist when /environmental_sample exists; where /environmental_sample exists, /isolation_source must exist. (Note that in Feature Table language, /environmental_sample refers to a sequence which has been obtained from a sample of mixed organisms where the individual organisms have not been isolated or identified prior to sequencing—see http://www.ebi.ac.uk/ena/WebFeat/qualifiers/environmental_sample.html). Syntactic validation also includes checking legal values of controlled vocabularies. For example, the /country qualifier uses a controlled list (http://www.insdc.org/country.html); the values of this qualifier are already constrained in web form views of the Annotation Checklists but cannot be controlled in user-built records, thereby requiring validation at the point of submission. The rule-base also contains an ever-growing class of biological rules, many of which depend on the taxonomic placement of the source organism. For example, /organelle can only exist where /organism belongs to Eukaryota, and the /transl_table for coding sequences must agree with the source organism's systematic range.

The ENA flat file Validator has now been made publicly available through the EBI website (http://www.ebi.ac.uk/ena/software/flat-file-validator). This enables users to check and amend their feature table annotation prior to submission, which is particularly useful in cases of large-scale annotation and entry uploads.

### Role of biocurator

The traditional role of the ENA biocurator has been to check the biological context of sequence data at the point of submission or update of existing records. The checks are several, requiring a broad knowledge of molecular biology across a taxonomic spectrum that spans viroids through to higher eukaryotes. With knowledge and experience of a specific data type or marker sequence, the biocurator can consider the best choice of annotation, can provide a standard level of consistency and can enrich the biological record. Much of this work has been carried out through direct communication with the submitter, ensuring that the record is valid at the time of submission or update. Although the biocurator's goal is to smooth and regulate the interface between the archive and the submitter, ultimately, the accuracy of the sequence, metadata and functional annotation is always the responsibility of the data owner.

Over the last six years, the role of the ENA biocurator has been transformed in response to the ever heavier use of our submission services that new sequencing technologies and broadening application of sequencing bring. During this transformation, we have worked to retain our attention to the capture and validation of functional annotation through the development of rules and formally expressed standards (such as the Annotation Checklists) and the construction of management systems to support these. The growing numbers of Annotation Checklists available within Webin (see Table 1), and the ever-growing rule-base encapsulated within the ENA Validator, provide both ENA and its submitters with a consistent and standardised method of error checking and, ultimately, greater utility of data within ENA and faster turnaround times from submission to issue of accession number and, where requested, public release of data. Biocurators have already witnessed an average time-saving of one third on submissions which arrive through Annotation Checklists compared to previously-available routes. In addition, genome assembly submissions no longer require any manual intervention assuming they successfully pass the automatic ENA validation step.

**Figure 2.**
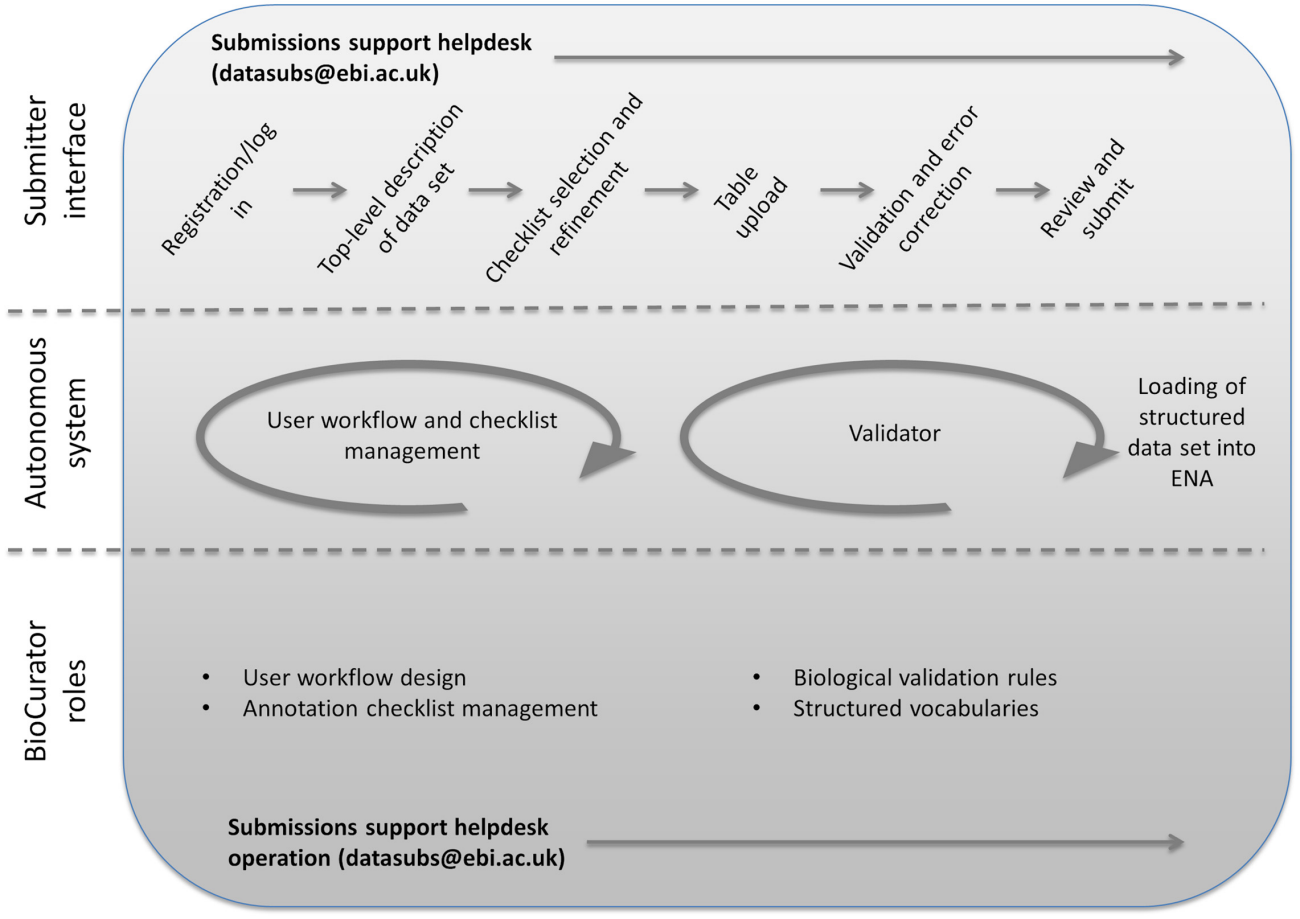
Submission workflow for functional annotation submissions under sustainable biocuration. The top level shows the flow through the submitter interface. The bottom level shows those biocurator roles which directly influence functional annotation submissions in ENA. Linking the work of the biocurator with the submitter interface is the autonomous system represented by the middle level.

As the landscape of genomics and sequencing changes, the role of the biocurator is thus adapting in a way which preserves the capacity to inject biological expertise into ENA content, while eliminating laborious record-by-record processing. A diagram showing the ultimate submitter workflow for sequence and functional annotation submission, and its relationship to sustainable biocuration, is shown in Figure 2.
